# Supplementary figures and images for: A hominoid-specific endogenous retrovirus may have rewired the gene regulatory network shared between primordial germ cells and naïve pluripotent cells
Source: PLoS Genet. 2022 May 12;18(5):e1009846. doi: 10.1371/journal.pgen.1009846 (PMC9128956; doi:10.1371/journal.pgen.1009846)

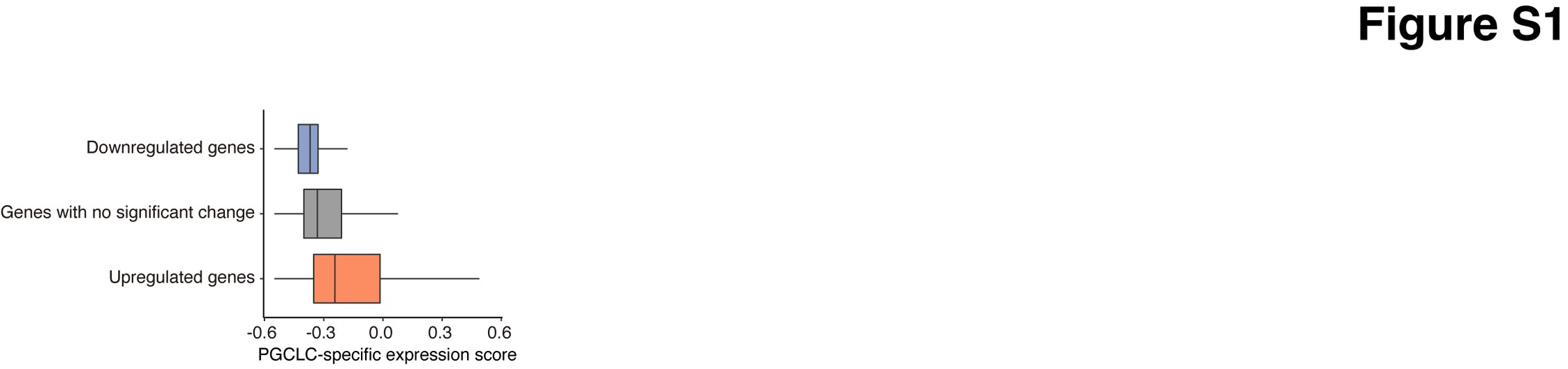

Supplement: S1 Fig — The scRNA-Seq data from Li et al. [8] was analyzed. As controls, the scores of genes that are downregulated in PGCs and those of genes that did not significantly change between PGCs and the later stages are shown. (TIF) [file pgen.1009846.s001.tif]

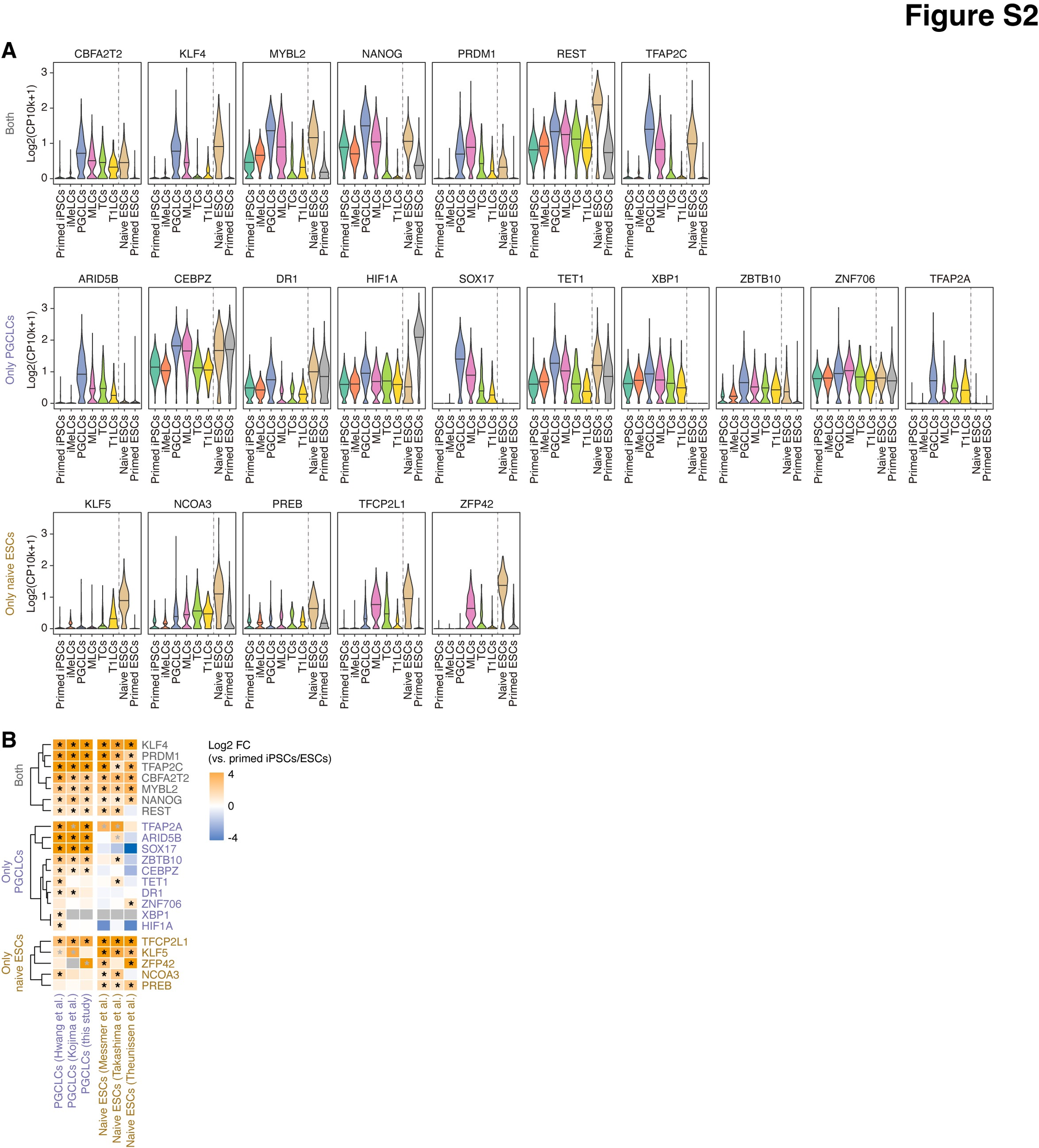

Supplement: S2 Fig — (A) Expression levels in various cell types from scRNA-Seq data for male germline development [Hwang et al. [10]] and for naïve and primed ESCs [Messmer et al. [36]]. The results for the TFs annotated in Fig 1C are shown. (B) Upregulation of TFs in PGCLCs and naïve ESCs observed across datasets. For the various datasets, the log2 FC values of the expression scores in PGCLCs vs. primed iPSCs or naïve ESCs vs. primed ESCs are shown. An asterisk denotes significant upregulation (FDR < 0.05; log2 FC > 1). A gray asterisk indicates that the expression level of the gene was not high (the mean expression level of the gene was below the 50th percentile for all expressed genes) even though significant upregulation was observed. For PGCLCs, the data of Hwang et al. [10] and Kojima et al. [12] were analyzed in addition to the original data in the present study. For naïve ESCs, the data of Messmer et al. [36], Takashima et al. [33], and Theunissen et al. [23] were analyzed. (TIF) [file pgen.1009846.s002.tif]

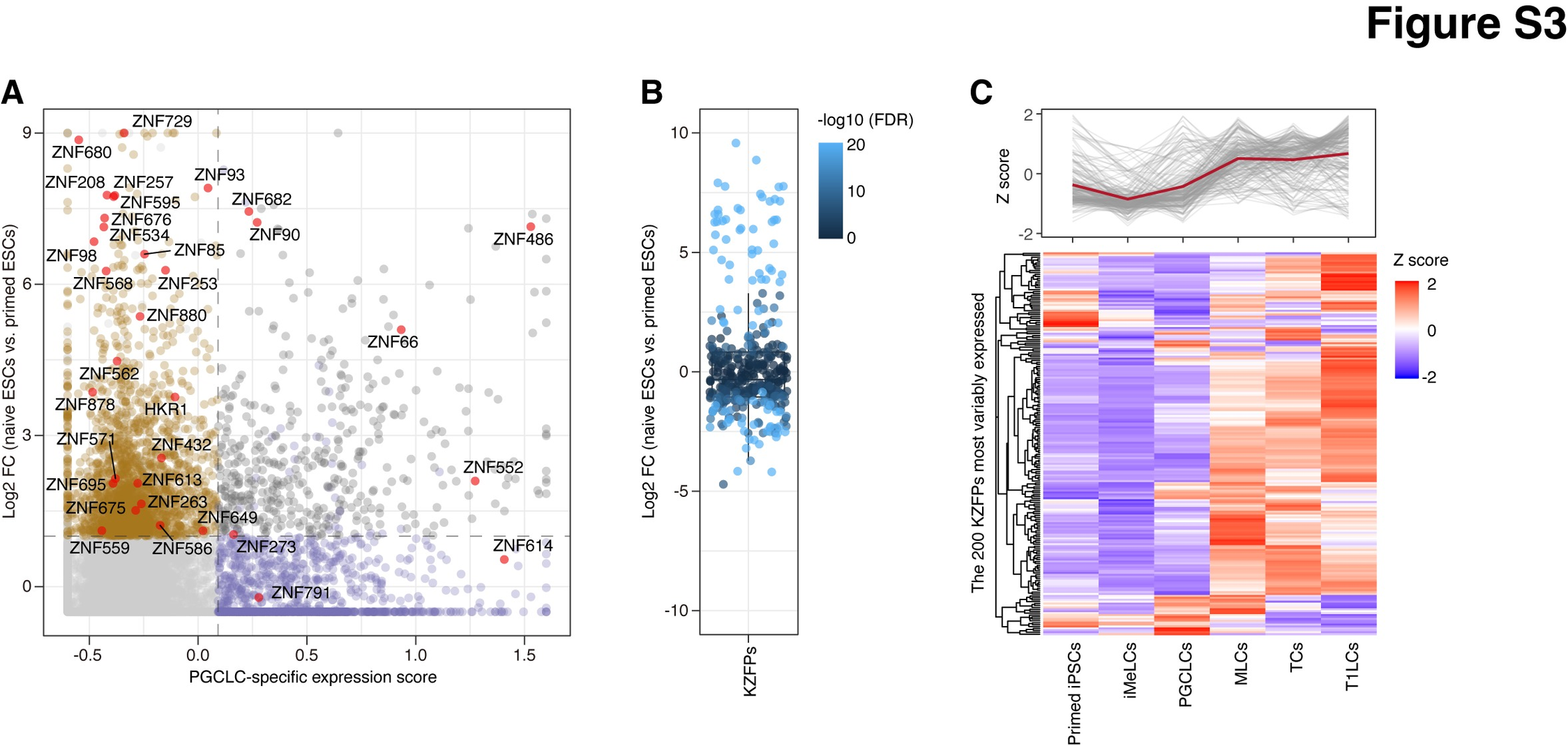

Supplement: S3 Fig — (A) Classification of KZFPs according to their expression patterns. Highly expressed KZFPs in PGCLCs or naïve ESCs are annotated. The results for TFs other than KZFPs are shown in Fig 1C. (B) Distributions of the log2 FC values of the expression scores of KZFPs in naïve ESCs vs. primed ESCs. The dot color denotes the statistical significance of the gene expression change. (C) Expression patterns of KZFPs during in vitro-derived human male germline development. The heatmap shows the relative mean expression values in the various cell types. The upper panel shows the transitions of the individual (gray) and mean (red) expression values. (TIF) [file pgen.1009846.s003.tif]

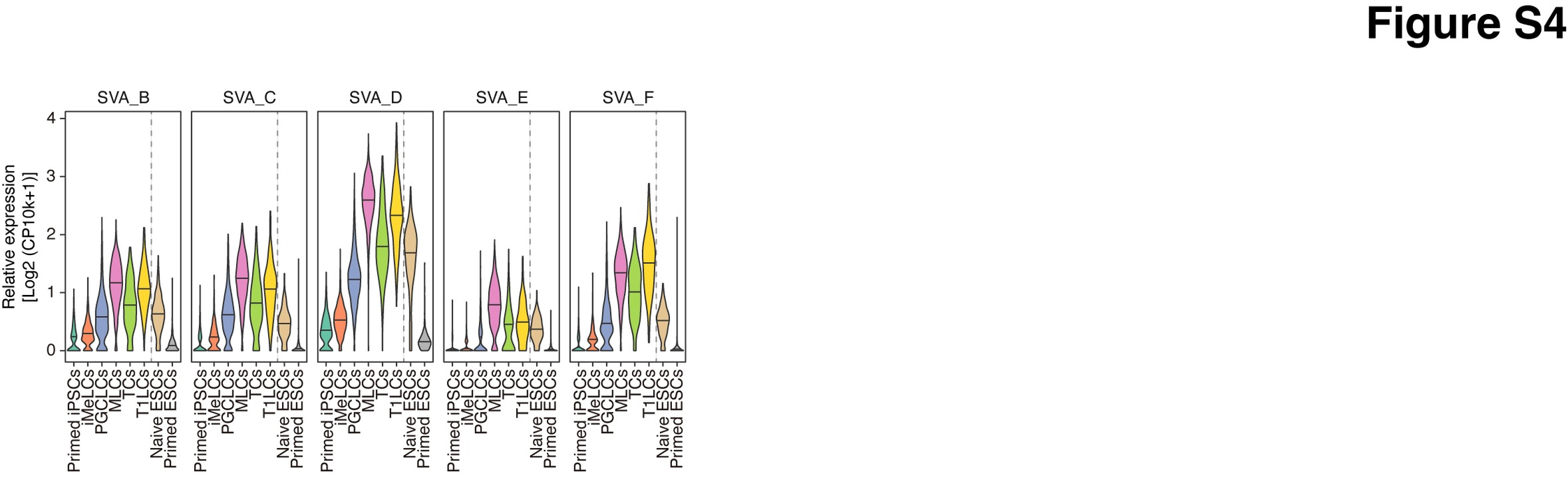

Supplement: S4 Fig — The results for the SVA transposons included in the heatmap in Fig 2B are shown. (TIF) [file pgen.1009846.s004.tif]

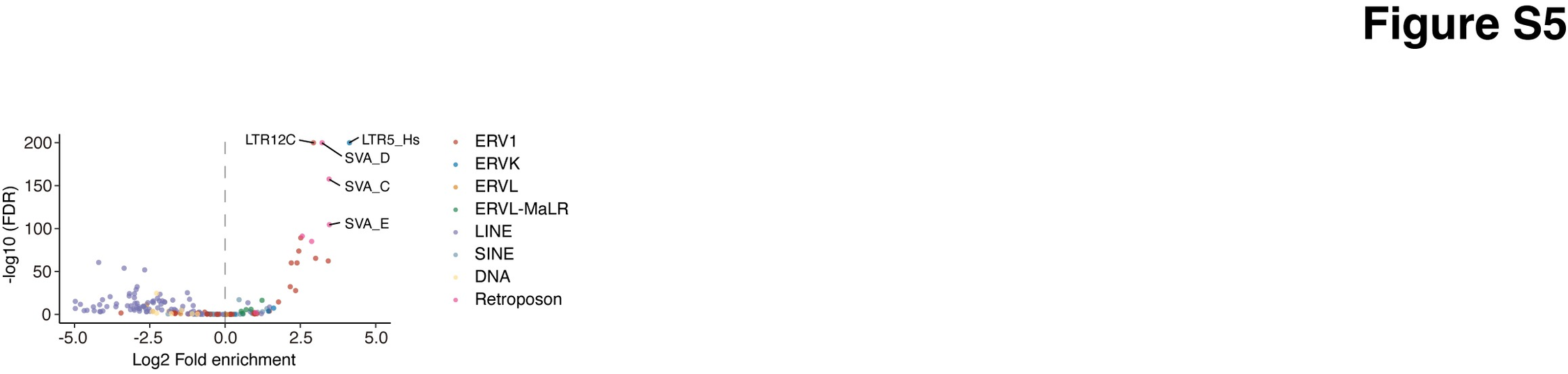

Supplement: S5 Fig — The ATAC-Seq data from Chen et al. [29] was analyzed. The log2 fold enrichment and statistical significance values are shown as a volcano plot. (TIF) [file pgen.1009846.s005.tif]

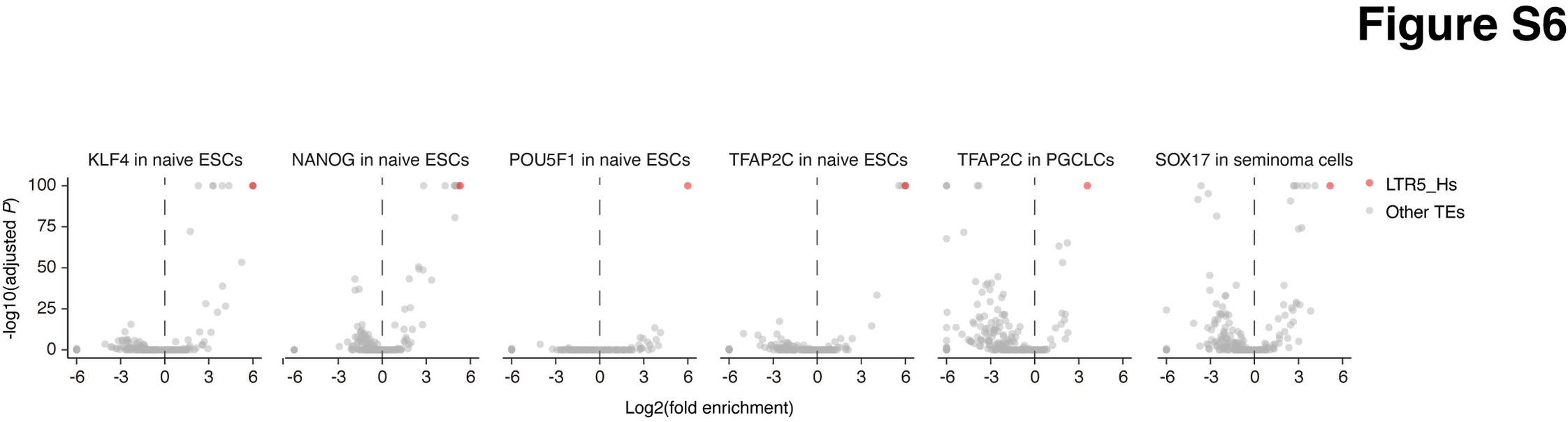

Supplement: S6 Fig — The enrichment for KLF4, NANOG, POU5F1, TFAP2C in naïve human ESCs and that for TFAP2C in PGCLCs are shown. In addition, the enrichment for SOX17 in a seminoma cell line (TCam-2 cells) is shown. The log2 fold enrichment and statistical significance scores are shown as a volcano plot. As controls, the enrichment scores for Tes other than LTR5_Hs are shown. (TIF) [file pgen.1009846.s006.tif]

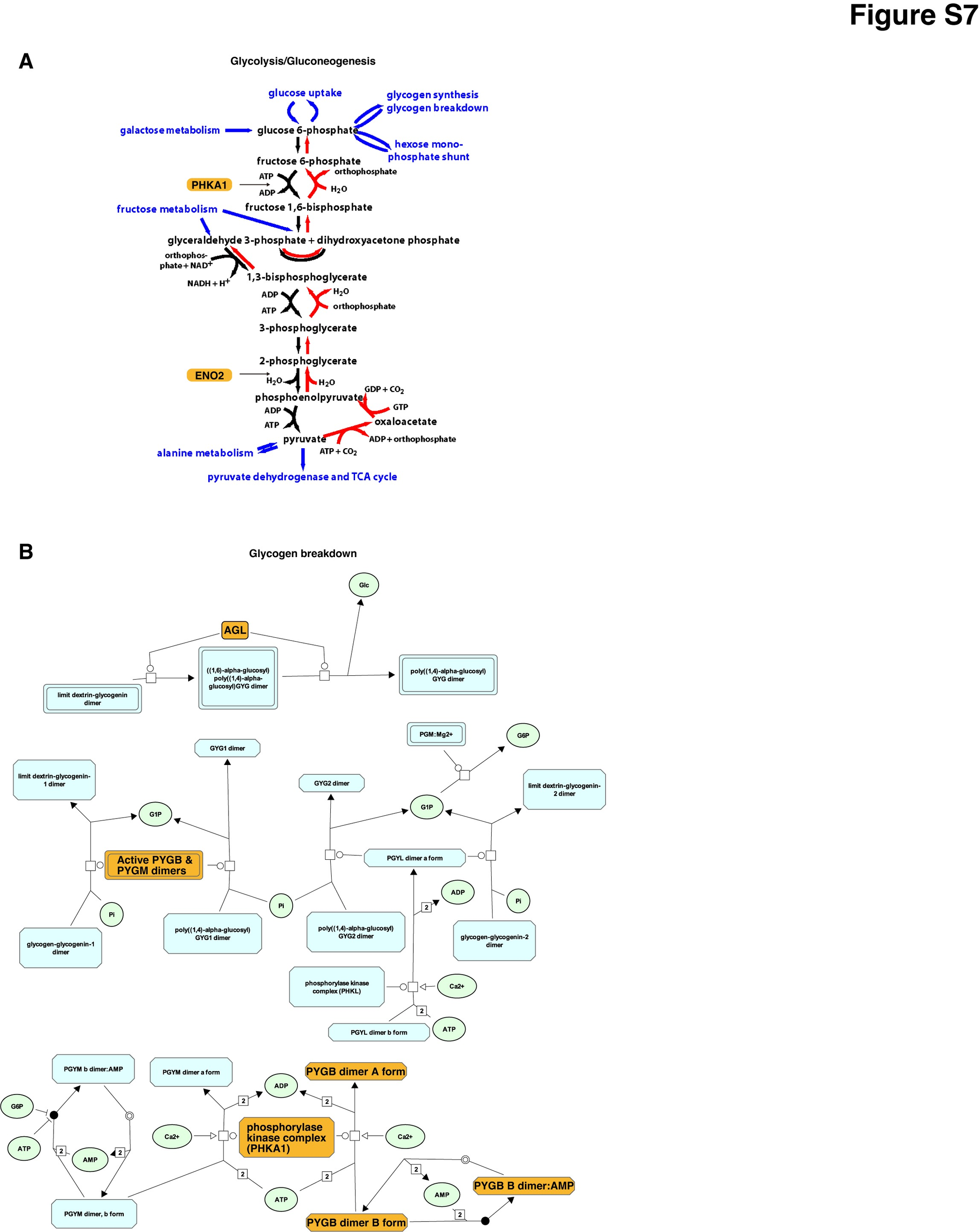

Supplement: S7 Fig — Pathway maps of glycolysis (A) and glycogen breakdown (B). Genes that are likely to be regulated by LTR5_Hs (i.e., AGL, ENO2, PFKL, PHKA1, and PYGB) are highlighted in orange. The pathway maps originated from the Reactome pathway database (https://reactome.org/) (65). (TIF) [file pgen.1009846.s007.tif]

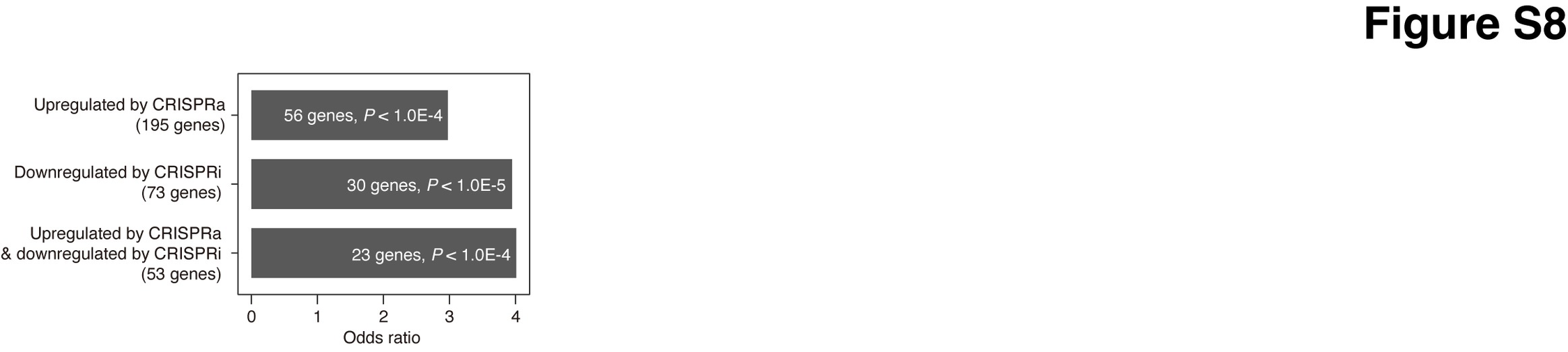

Supplement: S8 Fig — The 95 genes that are likely to be regulated by LTR5_Hs (shown in Fig 6E) were compared with genes that were up- or downregulated by CRISPRa or CRISPRi systems in embryonic carcinoma cells in a previous study (Fuentes et al.) [25]. To perform a fair comparison, only genes contained in both datasets and located within 50 kb from LTR5_Hs insertions (447 genes) were included in this analysis. These genes were stratified according to (i) whether the genes were included in the 95 genes (shown in Fig 6E) and (ii) whether the genes were perturbed by CRISPR systems in Fuentes et al. [25] (adjusted p value < 0.05, log2 FC value > 1 for upregulation, log2 FC value < -1 for downregulation). Subsequently, the degree of overlap between the stratified gene sets was evaluated. The P value was calculated with Fisher’s exact test. Information on the genes is summarized in S7 Table. (TIF) [file pgen.1009846.s008.tif]

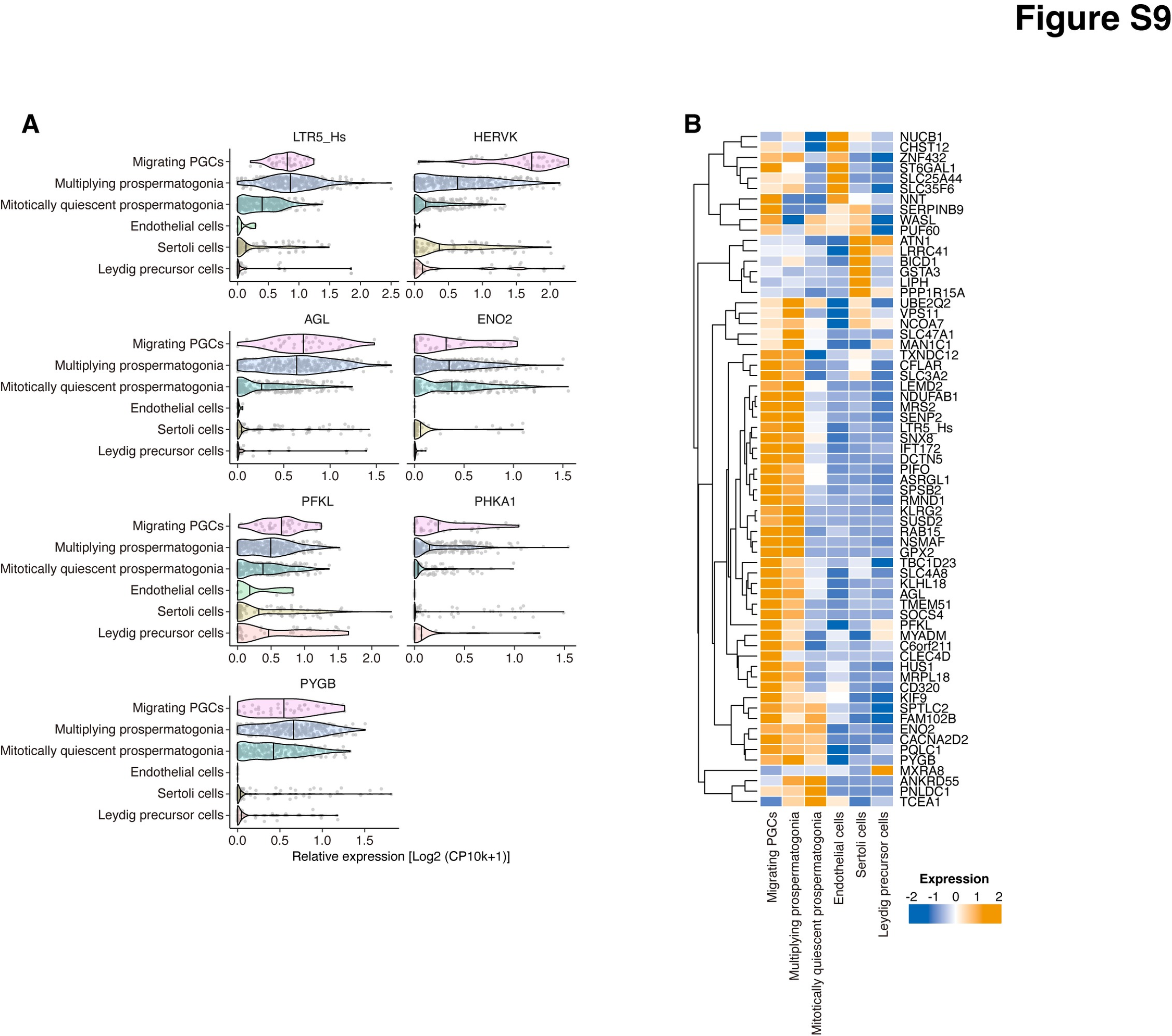

Supplement: S9 Fig — The scRNA-Seq data from Li et al. [8], which includes male germ cells (migrating PGCs and multiplying and mitotically quiescent prospermatogonia) and somatic cells at 4–25 weeks post-fertilization, was analyzed. Please note that migrating PGCs are more differentiated than the stage represented by PGCLCs (pre-migratory stage, ≤3 weeks post-fertilization). A) Violin plot showing the expression of LTR5_Hs, HERVK, and the glucose metabolism-related genes. B) Heatmap showing the normalized mean expression of genes that are likely regulated by LTR5_Hs (defined in Fig 6E). (TIF) [file pgen.1009846.s009.tif]

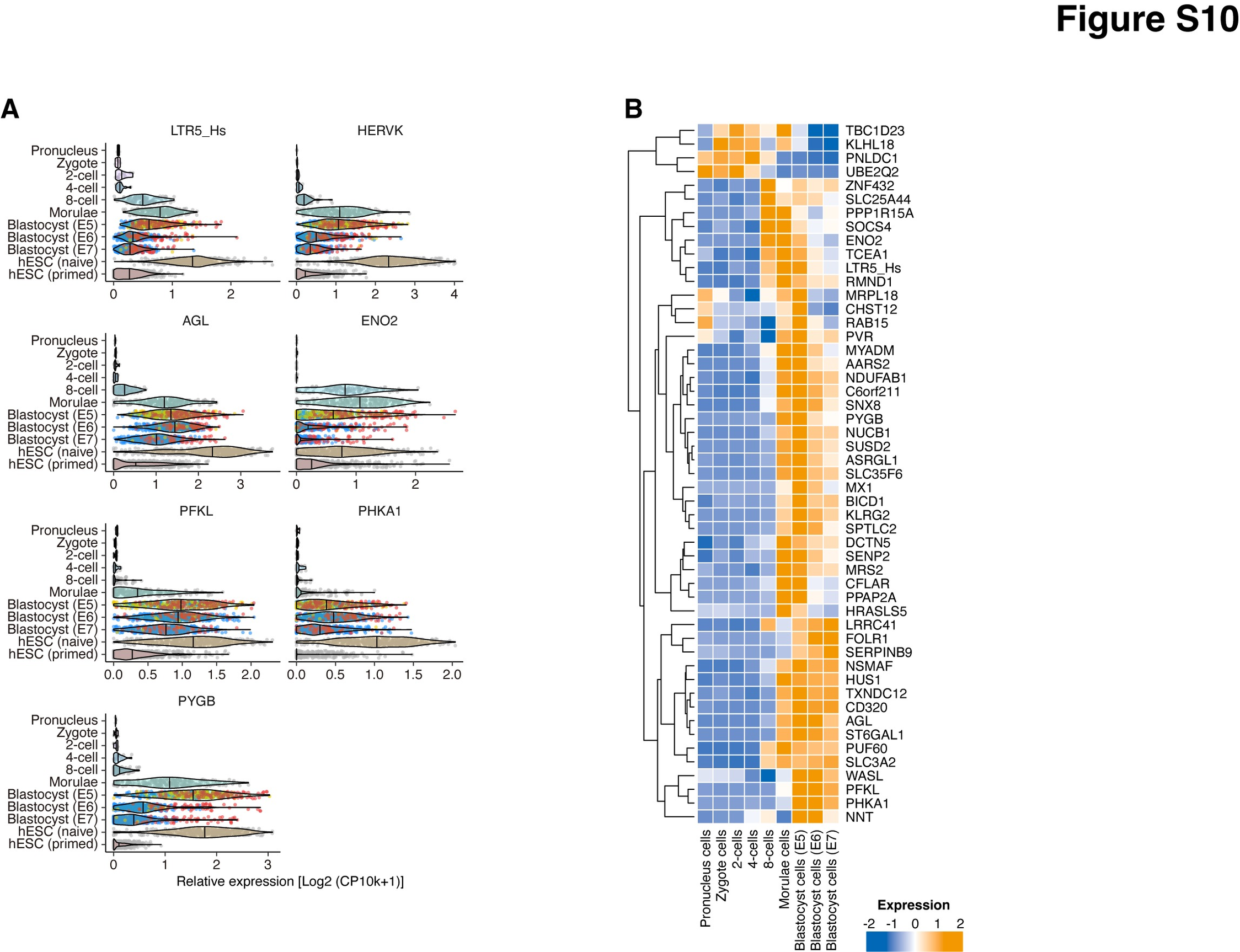

Supplement: S10 Fig — The scRNA-Seq datasets from various studies [48–50], which include from Pronucleus cells to blastocysts at embryonic day 7, were merged and analyzed. A) Violin plot showing the expression of LTR5_Hs, HERVK, and the glucose metabolism-related genes. In blastocysts, dots for ICM (red), trophectoderm (blue), and pre-lineage (yellow) are colored. In addition to data for the embryonic cells described above, data for naïve and primed ESCs [36] are shown. B) Heatmap showing the normalized mean expression of genes that are likely regulated by LTR5_Hs (defined in Fig 6E). (TIF) [file pgen.1009846.s010.tif]

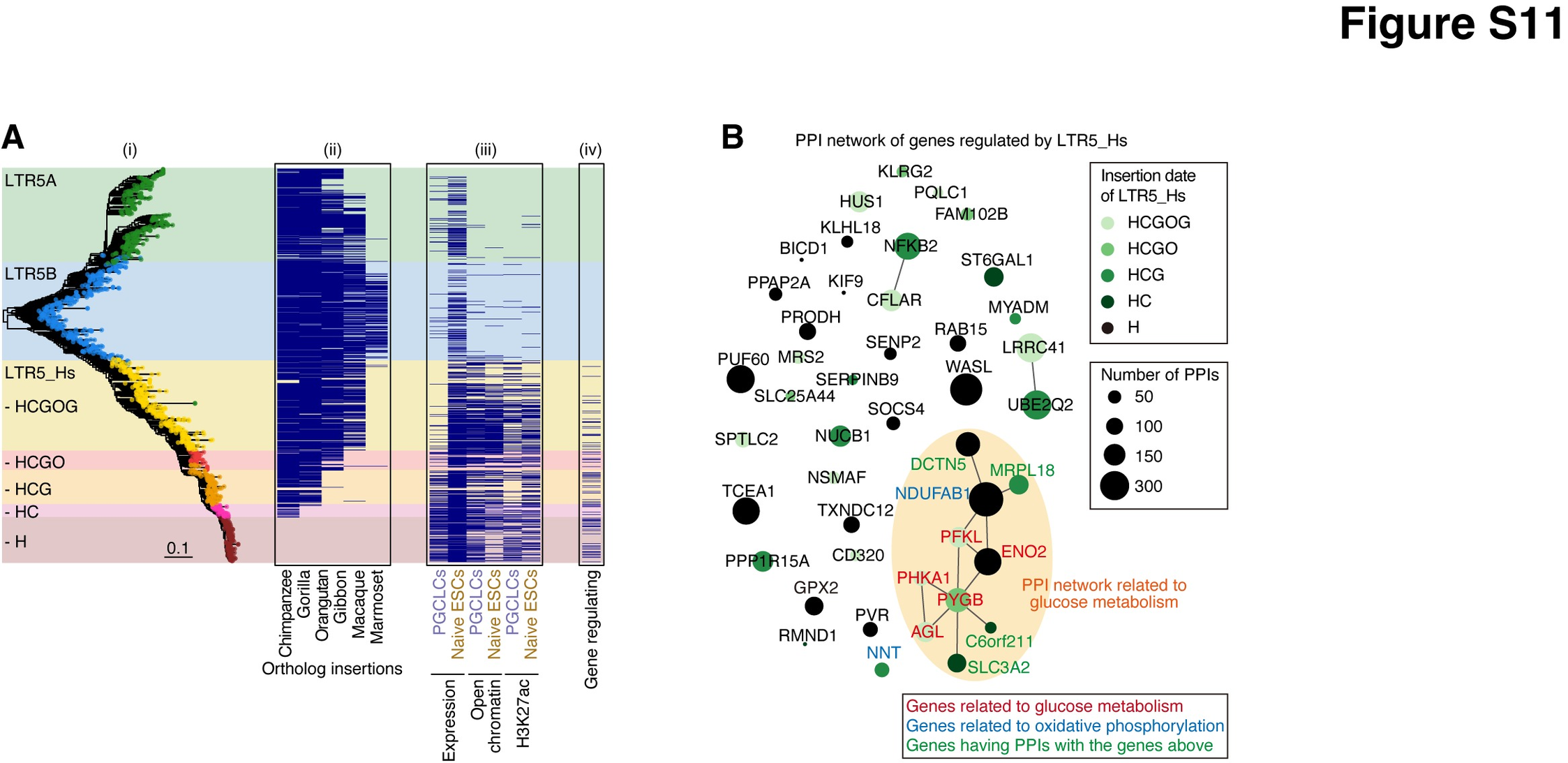

Supplement: S11 Fig — (A) Stratification of LTR5_Hs loci in the human genome according to their insertion dates. (i) Phylogenetic tree of the LTR5 family (including LTR5_Hs and related subfamilies [i.e., LTR5A and LTR5B]). (ii) Information on the distribution of orthologous insertions of LTR5 loci among primate genomes. According to the ortholog distribution and phylogeny, LTR5_Hs loci were stratified into five categories (HCGOG, HCGO, HCG, HC, and H). (iii) Epigenetic and transcriptomic statuses of various LTR5_Hs loci. (iv) LTR5_Hs loci that are likely to be associated with gene regulation. (B) PPI network for the genes likely to be regulated by LTR5_Hs. Only PPI links among the proteins encoded by the displayed genes are shown. The node color denotes the insertion date of the associated LTR5_Hs of the gene. The node size is proportional to the number of interacting partners in the whole PPI network. The glucose metabolism-related network is circled in orange. The PPI information originated from the STRING database [72]. (TIF) [file pgen.1009846.s011.tif]

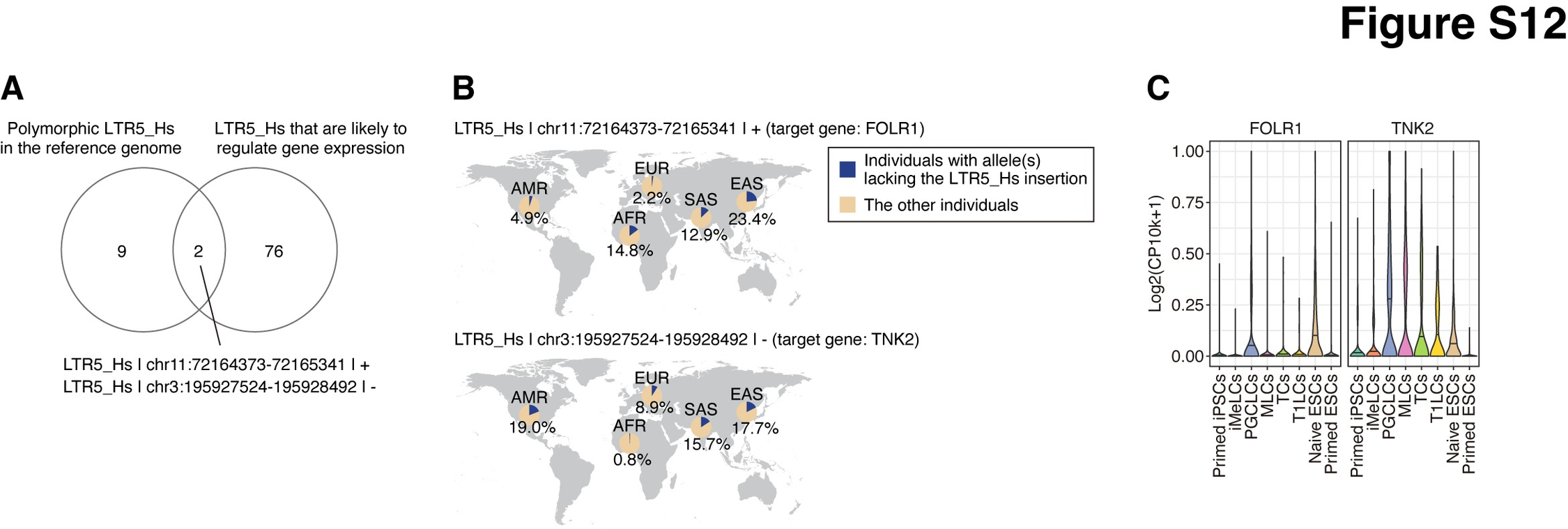

Supplement: S12 Fig — LTR5_Hs loci that are present in the human reference genome but not fixed in the human population (referred to as polymorphic LTR5_Hs loci) were identified using 1000 Genome Project datasets [52]. Information on the polymorphic LTR5_Hs loci is summarized in S9 Table. (A) Comparison of the polymorphic LTR5_Hs loci and the LTR5_Hs loci that are likely to regulate the gene expression in PGCLCs and naïve ESCs. The names of the overlapping LTR5_Hs loci are shown ("LTR5_Hs|chr11:72164373–72165341|+" and "LTR5_Hs|chr3:195927524–195928492|-"). (B) Geographical prevalence of the polymorphic LTR5_Hs loci in different human populations. Proportions of individuals with allele(s) lacking the LTR5_Hs insertion in different populations are shown. AFR, African; AMR, Ad Mixed American; EAS, East Asian; EUR, European; and SAS, South Asian. The map was generated using R maps (https://cran.r-project.org/web/packages/maps/index.html). (C) Expression levels of the genes associated with polymorphic LTR5_Hs in various cell types. (TIF) [file pgen.1009846.s012.tif]

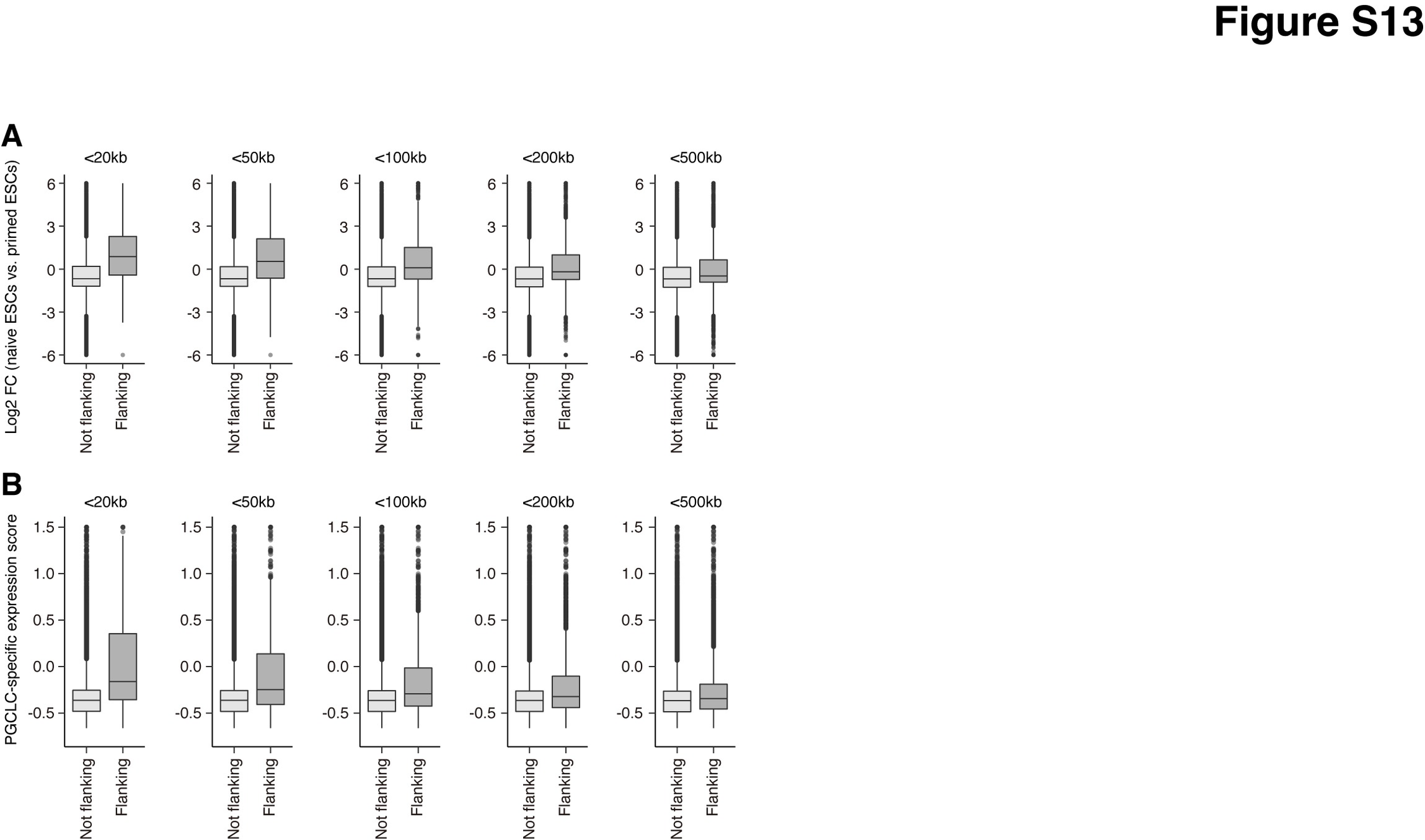

Supplement: S13 Fig — Protein-coding genes were classified into genes adjacent to LTR5_Hs or not according to the various distance thresholds for proximity definition. Subsequently, Log2 FC value (naïve ESCs vs. primed ESCs) and PGCLC-specific expression score were compared between the two gene categories. (TIF) [file pgen.1009846.s013.tif]
